# Genome-Wide Identification and Expression Analysis of SWEET Family Genes in Sweet Potato and Its Two Diploid Relatives

**DOI:** 10.3390/ijms232415848

**Published:** 2022-12-13

**Authors:** Zhuoru Dai, Pengyu Yan, Shaozhen He, Licong Jia, Yannan Wang, Qingchang Liu, Hong Zhai, Ning Zhao, Shaopei Gao, Huan Zhang

**Affiliations:** 1 Key Laboratory of Sweet Potato Biology and Biotechnology, Ministry of Agriculture and Rural Affairs/Beijing Key Laboratory of Crop Genetic Improvement/Laboratory of Crop Heterosis & Utilization and Joint Laboratory for International Cooperation in Crop Molecular Breeding, Ministry of Education, College of Agronomy & Biotechnology, China Agricultural University, Beijing 100193, China; 2 Sanya Institute, China Agricultural University, Sanya 572025, China; 3 Institute of Grain and Oil Crops, Yantai Academy of Agricultural Sciences, Yantai 265500, China; 4 Cereal Crops Research Institute, Henan Academy of Agricultural Sciences, Zhengzhou 450002, China

**Keywords:** sweet potato, *SWEET*, tissue-specific expression, tuberous root development, hormone treatment, abiotic stress

## Abstract

Sugar Will Eventually be Exported Transporter (SWEET) proteins are key transporters in sugar transportation. They are involved in the regulation of plant growth and development, hormone crosstalk, and biotic and abiotic stress responses. However, SWEET family genes have not been explored in the sweet potato. In this study, we identified 27, 27, and 25 SWEETs in cultivated hexaploid sweet potato (*Ipomoea batatas*, 2n = 6x = 90) and its two diploid relatives, *Ipomoea trifida* (2n = 2x = 30) and *Ipomoea triloba* (2n = 2x = 30), respectively. These SWEETs were divided into four subgroups according to their phylogenetic relationships with *Arabidopsis*. The protein physiological properties, chromosome localization, phylogenetic relationships, gene structures, promoter *cis*-elements, protein interaction networks, and expression patterns of these 79 *SWEETs* were systematically investigated. The results suggested that homologous SWEETs are differentiated in sweet potato and its two diploid relatives and play various vital roles in plant growth, tuberous root development, carotenoid accumulation, hormone crosstalk, and abiotic stress response. This work provides a comprehensive comparison and furthers our understanding of the SWEET genes in the sweet potato and its two diploid relatives, thereby supplying a theoretical foundation for their functional study and further facilitating the molecular breeding of sweet potato.

## 1. Introduction

Sugar Will Eventually be Exported Transporters (SWEETs) play key roles in sugar transport across plasma and intracellular membranes in both prokaryotes and eukaryotes [1]. Almost all SWEETs are present in the membrane structure, such as the plasma membrane and Golgi membrane [2]. As membrane proteins, SWEETs have three transmembrane domains (3TMs) in bacteria but have seven transmembrane domains (7TMs) in eukaryotes [3]. The 3TMs are encoded by a PQ-loop called the Mtn3 domain, which carries conserved proline and glutamine motifs [4,5]. The 7TM helices are folded into two parallel three-helix bundles connected by one central TM [1,6,7]. Since the 7TMs in SWEETs may not be sufficient for creating a functional pore as other types of sugar transporters carrying 12TMs, two SWEETs usually form a functional pore that permits sugar substrate transportation by oligomerization [1,3,7,8]. Accumulating evidence has revealed that SWEETs could homo- or hetero-oligomerize. The co-expression of a mutated and non-functional AtSWEET1 with a functional AtSWEET1 was found to inhibit sugar transport activity [9]. The oligomerization of the mutated form of OsSWEET11 with functional OsSWEET11 was found to disrupt sugar transport activity [10]. AtSWEET11 and AtSWEET12 undergo hetero-oligomerization to form a functional pore for sucrose transportation [11]. The hetero-oligomerization of SUT1 and SUT2 was found to be involved in the negative regulation of sucrose transportation [12].

In plants, the number of SWEETs varies among different species. The *Arabidopsis*, rice, potato, and soybean genomes encode 17, 21, 35, and 52 SWEETs, respectively [9,13,14,15]. These are critical in organ formation due to their controlling sugar transport [9,16]. In *Arabidopsis*, AtSWEET11, AtSWEET12, and AtWEET15 are important transporters for seed filling [17,18]*. AtSWEET11* and *AtSWEET12* are highly expressed in leaf phloem parenchyma cells, and the mutations of *AtSWEET11* and *AtSWEET12* result in defects in phloem loading [19]. Under dark or fructose accumulation, AtSWEET17, as a facilitator, was found to regulate the flow of fructose in vacuoles [16]. Mutations to *StSWEET11* were found to cause sucrose accumulation in leaves, leading to yield reductions in potato [20]. The overexpression of *PbSWEET4* caused reductions in sugar and early senescence in leaves in pears [21]. Moreover, SWEETs are also involved in the regulation of plant growth and development and hormone response. *AtSWEET8* is necessary for pollen growth [22]. *GmSWEET10a* and *GmSWEET10b* directly affect seed qualities in soybean [23]. The *AtSWEET13* and *AtSWEET14* double-mutant line failed to transport exogenous GA [24]. The rice *OsSWEET3a* was found to be involved in transporting glucose and gibberellin (GA) to leaves during early plant development [25]. The overexpression of *OsSWEET5* inhibited auxin concentration and signaling [26]. The triple mutants of *ZmSWEET13a, ZmSWEET13b*, and *ZmSWEET13c* resulted in a stunted phenotype in maize [27]. Furthermore, SWEETs are also involved in the regulation of biotic and abiotic stress responses. AtSWEET2 transports sugar from the cytosol to the vacuole, causing sugar leakage and thereby limiting pathogen growth [18]. The overexpression of *IbSWEET10* enhanced *Fusarium oxysporum* resistance by reducing the sugar content in the transgenic plants of the sweet potato [28]. *AtSWEET16* was found to enhance the freezing tolerance of transgenic plants [29]. Cucumber *CsSWEET2* was found to improve cold tolerance in *Arabidopsis* [30]. However, the biological functions and regulatory mechanisms of SWEETs remain unclear in sweet potato.

The sweet potato (*Ipomoea batatas* (L.) Lam., 2n = B_1_B_1_B_2_B_2_B_2_B_2_ = 6x = 90), belonging to the family Convolvulaceae, is an economically important root and tuber crop that is widely used as an industrial and bioenergy resource worldwide [31]. It provides a rich source of carbohydrates, dietary fiber, carotenoid, vitamins, and micronutrients. Due to its resilience and adaptability, it plays an important role in food security for subsistence farmers in Africa and Southeast Asia [31]. The formation and thickening of tuberous roots is one of the most important processes determining the yield of sweet potato. However, its diploids cannot form tuberous roots, and they exhibit slender stems and rattan characteristics [32,33,34]. In recent years, genome assemblies of a hexaploid sweet potato, Taizhong 6 [35], and two diploid species closely related to the hexaploid sweet potato, *Ipomoea trifida* NCNSP0306 (2n = 2x = 30) and *Ipomoea triloba* NCNSP0323 (2n = 2x = 30) [36], were released, making it possible to identify and analyze important gene families involved in tuberous root development at the whole-genome level in sweet potato.

In this study, SWEET family genes were identified from *I. batatas, Ipomoea trifida*, and *Ipomoea triloba*. We systematically investigated the protein physicochemical properties, chromosome localization, phylogenetic relationships, gene structure, *cis*-elements of promoters, and the protein interaction network of SWEETs in sweet potato. In addition, the tissue specificity and expression pattern analyses for tuberous root development in different varieties, and hormone responses (in leaves) of SWEETs were carried out using qRT-PCR and RNA-seq. The results play an important guiding role in the further study of their functions and the molecular breeding of the sweet potato.

## 2. Results

### 2.1. Identification and Characterization of SWEETs in the Sweet Potato and Two Diploid Relatives

**Figure 1:**
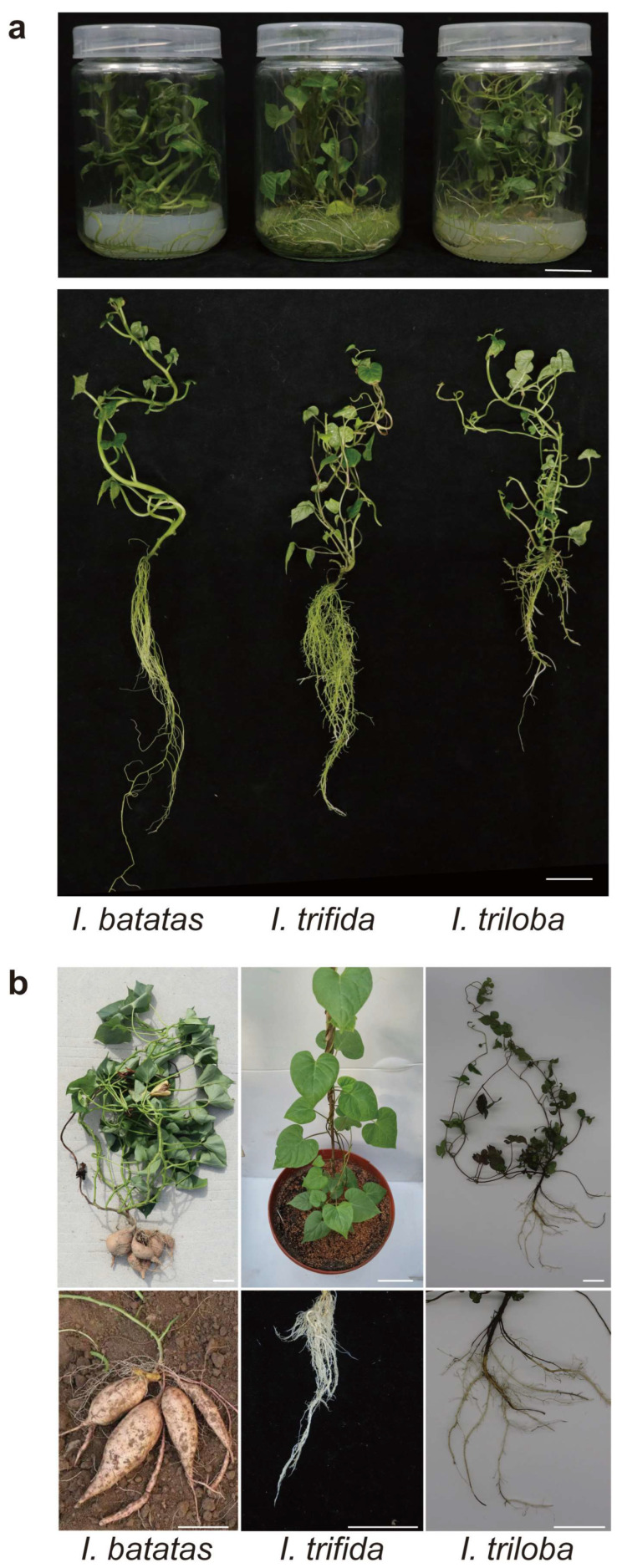
Plant morphology of in vitro grown (**a**) and field-grown plants. Scale bars, 2 cm. (**b**) of *I. batatas, I. trifida*, and *I.triloba*. Scale bars, 5 cm.

The plant morphology of the cultivated hexaploid sweet potato is different from that of its diploid relatives, especially since the diploid relatives cannot form tuberous roots (Figure 1). To comprehensively identify all SWEETs in the sweet potato and its two diploid relatives, we employed three typical strategies (i.e., blastp search, hmmersearch, and the CD-search database). A total of 79 SWEETs were identified in *I. batatas* (27), *I. trifida* (27), and *I. triloba* (25), which were named “*Ib*”, “*Itf*”, and “*Itb*”, respectively. The physicochemical properties were analyzed using the sequence of IbSWEETs (Table 1). The genomic length of the 27 *IbSWEETs* ranged from 1052 bp (*IbSWEET8.1*) to 5747 bp (IbSWEET15.7), and the CDS length varied from 823 bp (*IbSWEET9.1*) to 1557 bp (*IbSWEET2.3*). The amino acid lengths of IbSWEETs ranged from 153 aa (IbSWEET15.7) to 321 aa *(IbSWEET15.1*), with the molecular weight (MW) varying from 17.64 kDa (IbSWEET15.7) to 35.41 kDa (IbSWEET15.1). The isoelectric point (pI) of IbSWEET15.6 (5.81) was the lowest among all the IbSWEETs, indicating that it is an acidic protein. The pI of the other SWEETs was distributed from 7.61 (IbSWEET15.1) to 9.98 (IbSWEET8.3), suggesting that they are basic proteins. All the IbSWEETs contained Ser, Thr, and Tyr phosphorylation sites. All the IbSWEETs were stable with an aliphatic index of more than 100, except for IbSWEET3.1, which obtained an aliphatic index of 98.25. The grand average of the hydropathicity (GRAVY) value of all the IbSWEET proteins varied from 0.281 (IbSWEET3.1) to 1.070 (IbSWEET2.3), indicating that they are hydrophobic. The subcellular localization prediction assay showed that most of IbSWEETs were located in the cell membrane, except three IbSWEETs: IbSWEET15.6 and IbSWEET15.7, which were located in the cell membrane and chloroplasts, and IbSWEET1.1, which was located in the cell membrane and Golgi apparatus. Most of the IbSWEETs have seven transmembrane helical segments (TMHs); several (i.e., IbSWEET6.3, -8.1, -8.3, -9.2, -9.3, -15.2, -15.3, -15.4, and -15.7) have six TMHs; a few (i.e., IbSWEET2.3, -3.1, -6.2, and -10.5) have five TMHs, and IbSWEET15.6 has four TMHs. The three-dimensional structural models showed that there are three conserved α-helices in both N-terminal and C-terminal of all IbSWEETs (Figure S1). 

**Figure 2:**
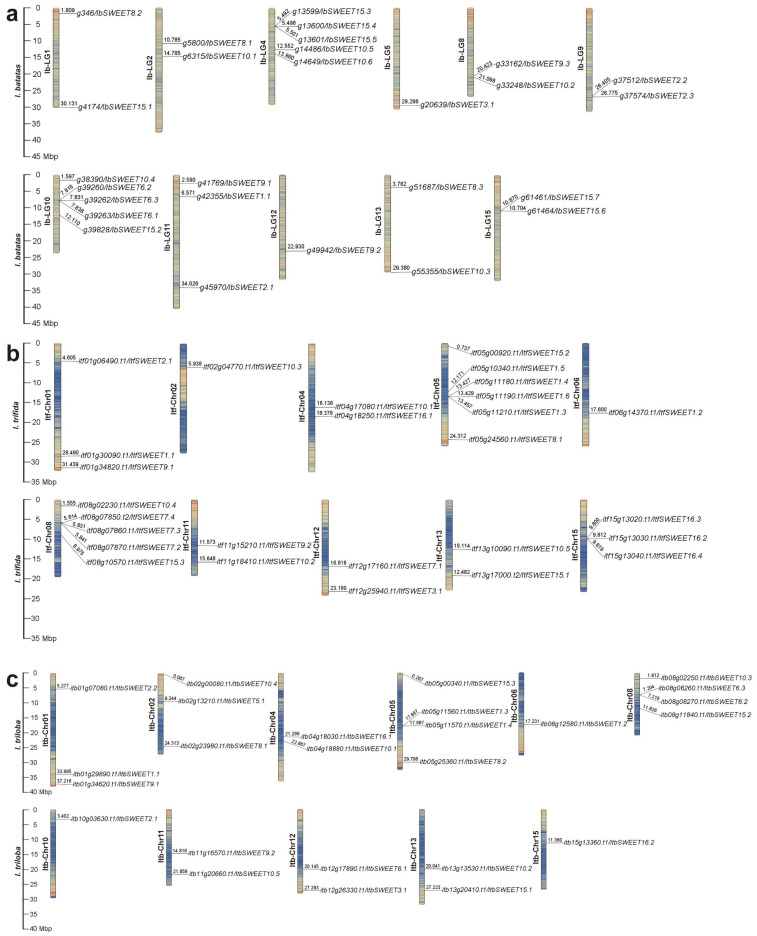
Chromosomal localization and distribution of *SWEETs* in *I. batatas* (**a**), *I. trifida* (**b**), and *I. triloba* (**c**). The bars represent chromosomes. The chromosome numbers are displayed on the left side, and the gene names are displayed on the right side. Each gene location is shown on the line. Detailed chromosomal location information is listed in Table S1.

The *SWEETs* were distributed across 11, 10, and 11 chromosomes of *I. batatas, I. trifida,* and *I. triloba*, respectively (Figure 2). In *I. batatas*, five *IbSWEETs* were detected on LG4 and LG10; three on LG11; two on LG1, LG2, LG8, LG9, LG13, and LG15; and one on LG5 and LG12, whereas no genes were detected on LG3, LG6, LG7, or LG14 (Figure 2a). In *I. trifida* and *I. triloba*, the distribution of *SWEETs* on Chr01 (3), Chr04 (2), Chr11 (2), Chr12 (2), Chr13 (2), and Chr06 (1) was similar, but their distribution on other chromosomes was different (Figure 2b,c). The results indicated a variation and loss of *SWEETs* during evolution, causing the difference between the distribution and disproportion of *SWEETs* on the chromosomes in sweet potato and its two diploid relatives.

### 2.2. Phylogenetic Relationship of SWEETs in the Sweet Potato and Its Two Diploid Relatives

**Figure 3:**
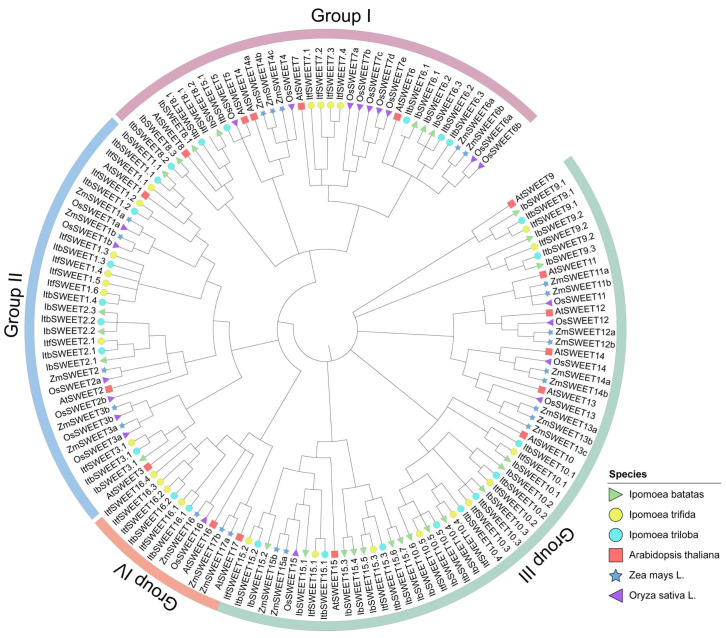
Phylogenetic analysis of the SWEET proteins from seven plant species (i.e., *I. batatas, I. trifida, I. triloba, Arabidopsis thaliana, Oryza sativa* L., and *Zea mays*). A total of 142 SWEETs were divided into four subgroup*s* (GroupⅠ to Group Ⅳ) according to the evolutionary distance. The green triangle, yellow circles, blue circles, red squares, purple triangle, and blue star represent the 27 IbSWEETs in *I. batatas*, 27 ItfSWEETs in *I. trifida*, 25 ItbSWEETs in I*. triloba,* 17 AtSWEETs in *Arabidopsis thaliana,* 21 OsSWEETs in *Oryza sativa* L., and 24 ZmSWEETs in *Zea mays,* respectively.

To study the evolutionary relationship of SWEETs in *I. batatas, I. trifida, I. triloba*, and *Arabidopsis*, we constructed a phylogenetic tree for 96 SWEETs of these four species (i.e., 27 in *I. batatas*, 27 in *I. trifida*, 25 in *I. triloba*, and 17 in *Arabidopsis*) (Figure 3). All the SWEETs were unevenly distributed on each branch of the phylogenetic tree. Interestingly, the SWEETs in *I. trifida, I. triloba*, and *Arabidopsis* were divided into four subgroups (Groups Ⅰ to Ⅳ), but in *I. batatas,* they were divided into three subgroups (Groups Ⅰ to Ⅲ) according to the evolutionary distance (Figure 3). The specific distribution of the SWEETs was as follows (total: *I.batatas, I. trifida, I. triloba*, and *Arabidopsis*): Group Ⅰ (22:6, 5, 6, 5), Group Ⅱ (23:5, 8, 7, 3), Group Ⅲ (43:16, 10, 10, 7), and Group Ⅳ (8:0, 4, 2, 2) (Figure 3; Table S1). We named IbSWEETs, ItfSWEETs, and ItbSWEETs based on their homology with homologs in *Arabidopsis*, and only AtSWEET1/2/3/5/6/7/8/9/10/15/16 from *Arabidopsis* had homologous proteins in *I. batatas, I. trifida,* and *I. triloba*. These results indicate that the number and type of SWEETs distributed in each subgroup in the sweet potato differed from those of its two diploid relatives and Arabidopsis.

Furthermore, a total of 142 SWEET proteins from six plant species (i.e., 27 in *I.batatas*, 27 in *I. trifida*, 25 in *I.triloba*, 17 in *Arabidopsis*, 21 in rice, and 24 in maize) were used for the phylogenetic analysis. They were divided into four subgroups (Groups Ⅰ to Ⅳ) (Figure 3), which indicated that the evolutionary relationship of the SWEETs was relatively conserved in the plant.

### 2.3. Conserved Motif and Exon–Intron Structure Analysis of SWEETs in the Sweet Potato and Two Diploid Relatives

**Figure 4:**
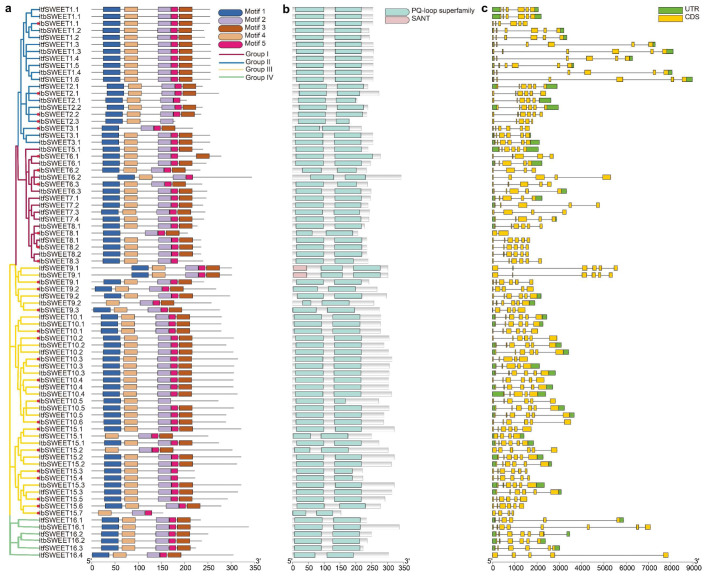
Conserved motifs and exon–intron structure analysis of the SWEET family in *I. batatas, I. trifida*, and, *I. triloba.* (**a**) The phylogenetic tree shows that SWEETs are distributed in four subgroups on the left, and the five conserved motifs are shown in different colors. The red circle represents the IbSWEETs. (**b**). Conserved domain structures of SWEETs. The blue box represents the PQ-loop domain. The red box represents the SANT domain. (**c**) Exon–intron structures of SWEETs. The green boxes, yellow boxes, and black lines represent the UTRs, exons, and introns, respectively.

Furthermore, sequence motifs in the 27 *IbSWEETs*, 27 *ItfSWEETs*, and 25 *ItbSWEETs* were analyzed using the MEME website, and the five most conserved motifs were identified (Figure 4a and Figure S2). Most of the SWEETs contained these five conserved motifs, except for a few SWEETs that were differentiated in the number and species of motifs in *I.batatas, I.trifida,* and *I.triloba*, such as IbSWEET15.2 (containing motifs 2–5), ItfSWEET15.2 (containing motifs 1–5), and ItbSWEET15.2 (containing motifs 1–5) (Figure 4a). The PQ-loop acts as a key structure for the helix of the SWEETs [9]—the first PQ-loop contains motifs 1 and 4 and the second PQ-loop contains motifs 2, 3, and 5; additionally, all the SWEETs contain two PQ-loops (Figure 4b). Moreover, only ItfSWEET9.1 and ItbSWEET9.1 contain an SANT domain, which is involved in the regulation of flower development [37] (Figure 4b).

To better understand the structural diversity among SWEETs, the exon–intron structures were analyzed (Figure 4c). The number of exons in the SWEETs ranged from two to eight. In more detail, the SWEETs of Group I contained two to six exons; the SWEETs of Group II contained five or six exons; the SWEETs of Group III contained four to six exons; and the SWEETs of Group IV contained five to eight exons (Figure 4c). The exon–intron structures of some homologous SWEETs were different in *I. batatas* compared to those in *I. trifida* and *I. triloba*, such as *IbSWEET8.1* (containing two exons), *ItfSWEET8.1* (containing six exons), and *ItbSWEET8.1* (containing six exons) in Group Ⅰ, *IbSWEET9.2* (containing five exons) and *ItbSWEET9.2* (containing six exons) in Group Ⅲ, and *ItfSWEET16.1* (containing six exons), and *ItbSWEET16.1* (containing eight exons) in Group Ⅳ (Figure 4c). These results indicated that the SWEET family may have undergone a lineage-specific differentiation event in the sweet potato genome.

### 2.4. Cis-Element Analysis in the Promoter of IbSWEETs in Sweet Potato

**Figure 5:**
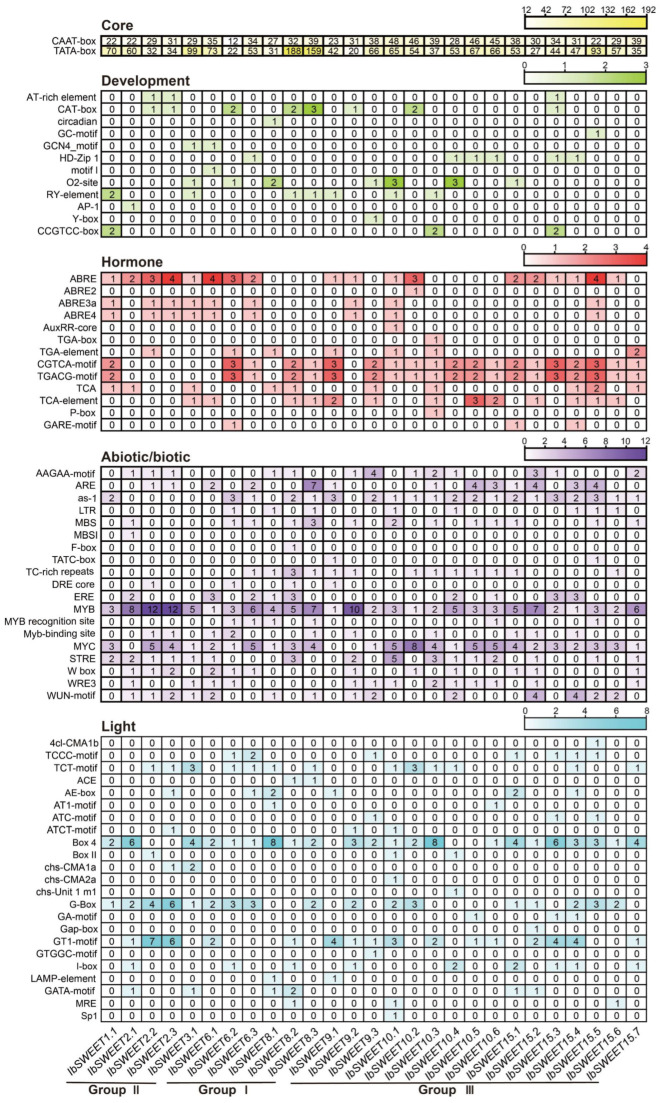
*Cis*-element analysis of *IbSWEET*s in *I. batatas*. The *cis*-elements were divided into five categories. The intensity of the different colors represents the number of *cis*-elements in the *IbSWEET* promoters.

Promoter c*is*-elements in plants initiate the gene functions related to plant development, hormone regulation, and stress response. Therefore, we performed a *cis*-element analysis using the 1500 bp promoter region of *IbSWEETs*. According to the predicted functions, we divided the elements into five categories: core elements, development regulation elements, hormone-responsive elements, abiotic/biotic stress-responsive elements, and light-responsive elements (Figure 5). A large number of core elements were identified in the 27 *IbSWEETs* (CAAT-box and TATA-box) (Figure 5). Most of the *IbSWEETs* contained several development elements, such as the O2-site, which was a zein metabolism regulatory element (found in *IbSWEET3.1,-6.2,-8.1,-9.3,-10.1,-10.4*, and *-15.1*); the CAT-box, which was associated with meristem formation (found in *IbSWEET2.2,-2.3,-6.2,-8.2,-8.3,-9.2,-10.2*, and *-15.3*); and the GCN4 motif, which was involved in controlling seed-specific expression (found in *IbSWEET3.1* and *IbSWEET6.1*) (Figure 5). However, no development-related elements were found in the 1500 bp promoter region of *IbSWEET15*.*2,IbSWEET15*.*6*, and *IbSWEET15*.*7*. Moreover, light-responsive elements such as the G-box, BOX4, and AE-box were abundant in the promoters of *IbSWEETs* (Figure 5).

Additionally, some abiotic elements, such as the drought-responsive elements DRE-core, MYB, and MYC; the salt-responsive elements LTR, MBS, and W-box; the light-responsive elements ERE and LTR; and biotic elements, such as WRE3, W-box, and the WUN motif, were identified in most *IbSWEETs* (Figure 5). All the *IbSWEETs* possessed several hormone elements, including ABRE for ABA-responsive elements, TGA-element for IAA-responsive elements, TATC-box for GA-responsive elements, the CGTCA and TGACG motifs for MeJA-responsive elements, and the TCA motif for SA-responsive elements (Figure 5). These results suggest that *IbSWEETs* are involved in the regulation of plant growth and development, hormone crosstalk, and abiotic stress adaption in the sweet potato.

### 2.5. Protein Interaction Network of IbSWEETs in the Sweet Potato

**Figure 6:**
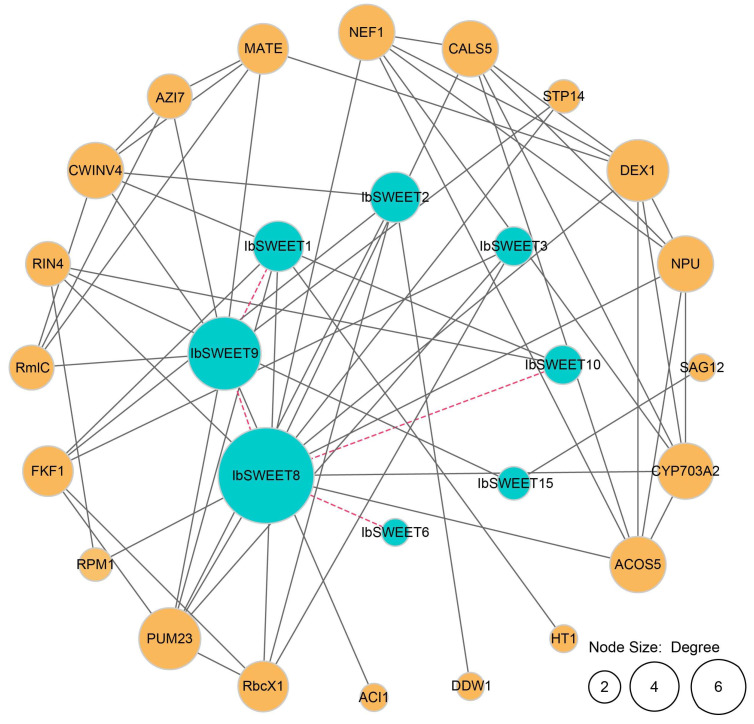
Functional interaction networks of IbSWEETs in *I. batatas* according to orthologues in *Arabidopsis.* Network nodes represent proteins, and lines represent protein–protein associations. The node size represents the number of proteins that interact with each other. The dotted line represents the interaction between the different SWEETs. The solid line represents the interaction between SWEETs and other proteins.

To explore the potential regulatory network of IbSWEETs, we constructed an IbSWEET interaction network based on *Arabidopsis* orthologous proteins (Figure 6). Protein interaction predictions indicated that some IbSWEETs (IbSWEET1, 6, 8, 9, and 10) could interact with other IbSWEETs to form heterodimers. In addition, SWEETs can interact with pollen development-related protein DEX1 [38], circadian rhythm-related protein FKF1 [39,40], and pathogen responsive-related protein RIN4 and RPM1 [41,42]. IbSWEET2, IbSWEET3, and IbSWEET9 can interact with translation regulation-related protein PUM23 [43]. IbSWEET15 can interact with plant senescence regulatory-related protein SAG12 [44]. These results indicate that IbSWEETs are involved in the regulation of plant growth and development and biotic stress adaption in the sweet potato.

### 2.6. Expression Analysis of SWEETs in the Sweet Potato and Two Diploid Relatives

#### 2.6.1. Expression Analysis in Various Tissues

**Figure 7:**
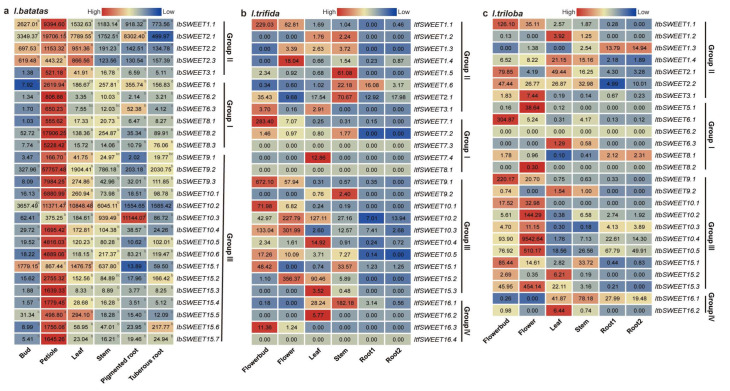
Gene expression patterns of SWEETs in different tissues of *I. batatas, I. trifida*, and *I. triloba.* (**a**) Expression analysis in the bud, petiole, leaf, stem, pencil root, and tuberous root of *I. batatas*. qRT-PCR determined the values from three biological replicates consisting of pools of three plants, and the results were analyzed using the comparative C_T_ method. The expression of *IbSWEET8.1* in the buds was considered as “1”. The fold change is shown in the boxes. Different lowercase letters indicate a significant difference in each *IbSWEET* at *p* < 0.05 based on the Student’s *t*-test. (**b**) Gene expression patterns of *ItfSWEETs* in the flower bud, flower, leaf, stem, root 1, and root 2 of *I. trifida* as determined by RNA-seq. The log_2_(FPKM) value is shown in the boxes. (**c**) Gene expression patterns of *ItbSWEETs* in the flower bud, flower, leaf, stem, root 1, and root 2 of *I. triloba* as determined by RNA-seq. The log_2_(FPKM) value is shown in the boxes.

To investigate the potential biological function of *IbSWEETs* in plant growth and development, the expression levels in six representative tissues (i.e., bud, petiole, leaf, stem, pencil root, and tuberous root) of *I. batatas* were analyzed using real-time quantitative PCR (qRT-PCR) (Figure 7). Nonetheless, different subgroups showed diversified expression patterns in six tissues. *IbSWEETs* in Group Ⅱ showed higher expression levels in all the tissues as compared to the other subgroups. Among all the *IbSWEETs*, six *IbSWEETs* (i.e., *IbSWEET1.1,−2.1,−2.2,−2.3,−9.2,* and *−10.2*) were highly expressed in all the tissues, especially *IbSWEET10.2*, which was highly expressed by more than 1000-fold in all the tissues. Interestingly, all the *IbSWEETs* showed high expression levels in the petiole. Moreover, some *IbSWEETs* showed tissue-specific expression—e.g., *IbSWEET1.1,-2.1,-2.2,-2.3*, and *−15.1* were highly expressed in buds; *IbSWEET2.1,-2.2,-2.3,-10.2*, and *-15.1* were highly expressed in leaves; *IbSWEET10.3* was highly expressed in stems and pencil roots; and *IbSWEET8.3* and *IbSWEET15.6* were highly expressed in tuberous roots (Figure 7a). These results indicate that *IbSWEETs* might play different roles in sugar transport and development in the various tissues of the sweet potato.

In addition, we used RNA-seq data of six tissues (i.e., flower bud, flower, leaf, stem, root1, and root2) to study the expression patterns of SWEETs in *I. trifida* and *I. triloba* [43] (Figure 7b,c). In *I. trifida, ItfSWEET1.1,-2.1, -7.1*,*- 9.1,-10.1,-10.3,-10.5,-15.1*, and *-16.3* were highly expressed in flowerbuds; *ItfSWEET1.1,-1.4,-9.1,-10.2,-10.3*, and *-15.2* were highly expressed in flowers; *ItfSWEET7.4,-10.2,-10.4,-15.2*, and *-16.2* were highly expressed in leaves; and *ItfSWEET1.5,-1.6,-2.1,-9.2,-15.1*, and *-16.1* were highly expressed in stems (Figure 7b). Almost all the *ItfSWEETs* had a low expression on levels in root1 and root2, except *ItfSWEET1.6* (16.08-fold in root1). In *I. triloba, ItbSWEET1.1,-2.1,-2.2,-6.1,-9.1*, and *-15.1* were highly expressed in flowerbuds; *ItbSWEET3.1,-5.1,-10.1,-10.2,-10.3,-10.4, -10.5*, and *-15.3* were highly expressed in flowers; *ItbSWEET1.2,-1.4,-2.1,-6.3,-9.2,-15.2*, and *-16.2* were highly expressed in leaves; *ItbSWEET2.2* and *ItbSWEET16.1* were highly expressed in stems; and *ItbSWEET1.3,-8.1*, and *-16.1* were highly expressed in roots (Figure 7c). These results showed that SWEETs exhibit different expression patterns and play important roles in the growth and development of the sweet potato and the two diploids.

#### 2.6.2. Expression Analysis in Different Developmental Stages

**Figure 8:**
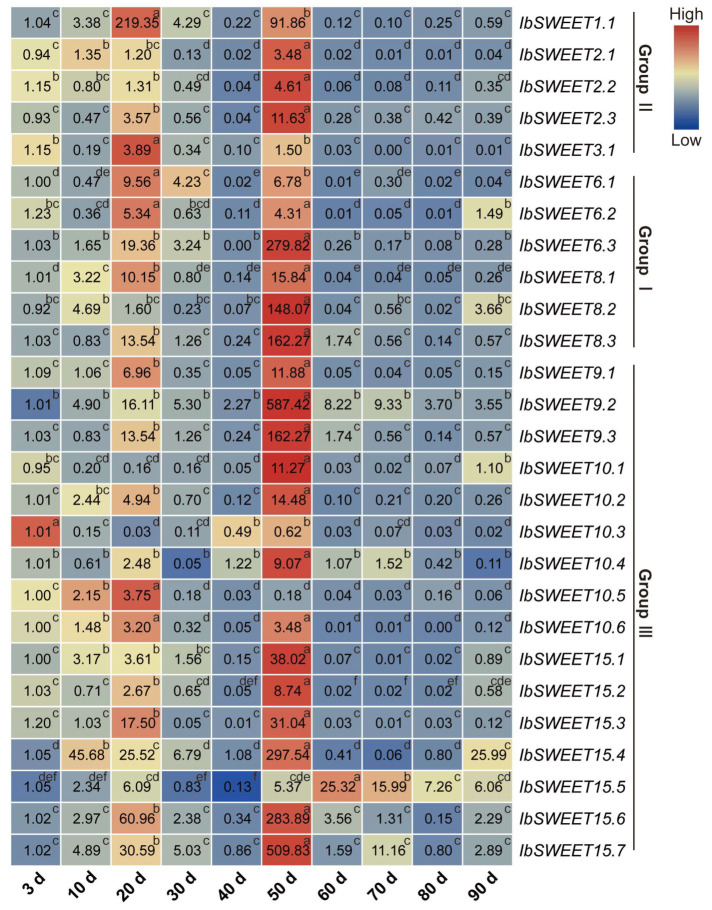
Gene expression patterns of *IbSWEETs* in different root developmental stages (i.e., at 3 d, 10 d, 20 d, 30 d, 40 d, 50 d, 60 d, 70 d, 80 d, and 90 d) as determined by qRT-PCR. The values were determined by qRT-PCR from three biological replicates consisting of pools of three plants, and the results (i.e., at 3 d, 10 d, 20 d, 30 d, 40 d, 50 d, 60 d, 70 d, 80 d, and 90 d) were analyzed using the comparative C_T_ method. The expression of 3 d was considered as “1”. The fold changes are shown in the boxes. Different lowercase letters indicate a significant difference of each *IbSWEET* at *p <* 0.05 based on Student’s *t*-test.

We further performed qRT-PCR to evaluate the expression levels of *IbSWEETs* in different developmental stages of sweet potato roots (i.e., at 3 d, 10 d, 20 d, 30 d, 40 d, 50 d, 60 d, 70 d, 80 d, and 90 d) (Figure 8). Notably, most *IbSWEETs* peaked at 20 d and 50 d, which were the initial development and the rapid expansion stage of tuberous roots, respectively. These results indicate that *IbSWEET*s are of vital importance to the growth and development of tuberous roots in the sweet potato.

#### 2.6.3. Expression Analysis in Different Varieties

**Figure 9:**
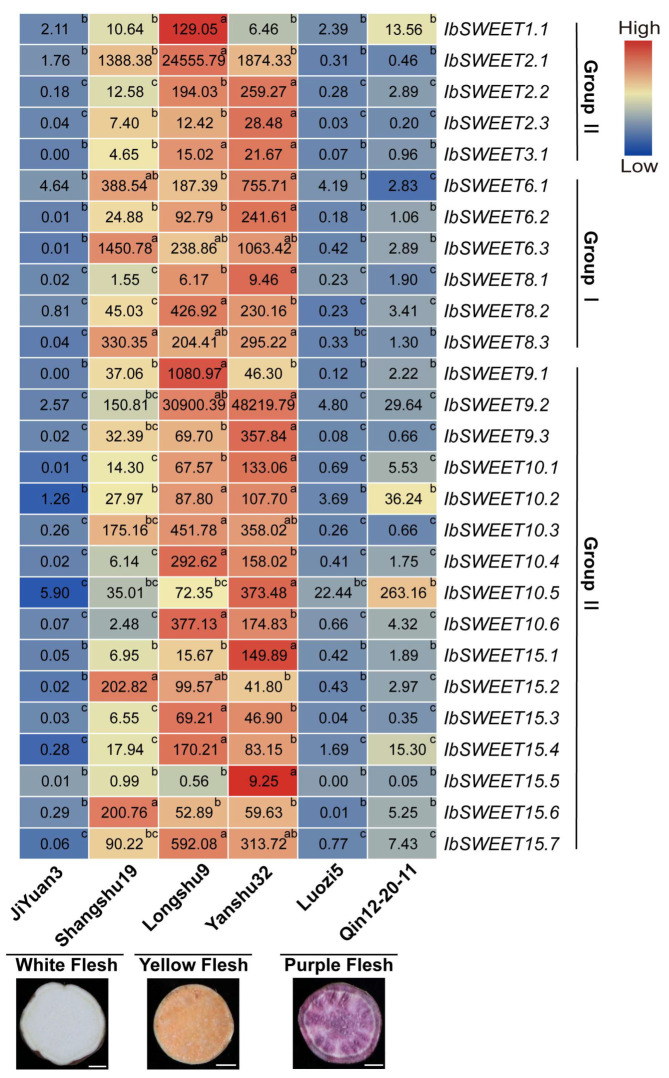
Gene expression patterns of *IbSWEETs* in different sweet potato varieties with different flesh colors. The values were determined by qRT-PCR from three biological replicates consisting of pools of three plants, and the results were analyzed using the comparative C_T_ method. The expression of *IbSWEET6.2* in Qin-12-20-11 was considered as “1”. The fold changes are shown in the boxes. Different lowercase letters indicate a significant difference of each *IbSWEET* at *p <* 0.05 based on Student’s *t*-test. Scale bars, 1 cm.

We analyzed the expression levels of *IbSWEETs* in sweet potato varieties with different flesh colors (white flesh: Jiyuan3 and Shangshu19; yellow flesh: Longshu9 and Yanshu32; purple flesh: Luozi5 and Qin12-20-11) (Figure 9). Interestingly, the expression levels of most *IbSWEETs* in the yellow-fleshed varieties were higher than those in the white- and purple-fleshed varieties. This data indicates that *IbSWEET*s may be involved in carotenoid accumulation in sweet potato tuberous roots.

#### 2.6.4. Expression Analysis of Hormone Response

**Figure 10:**
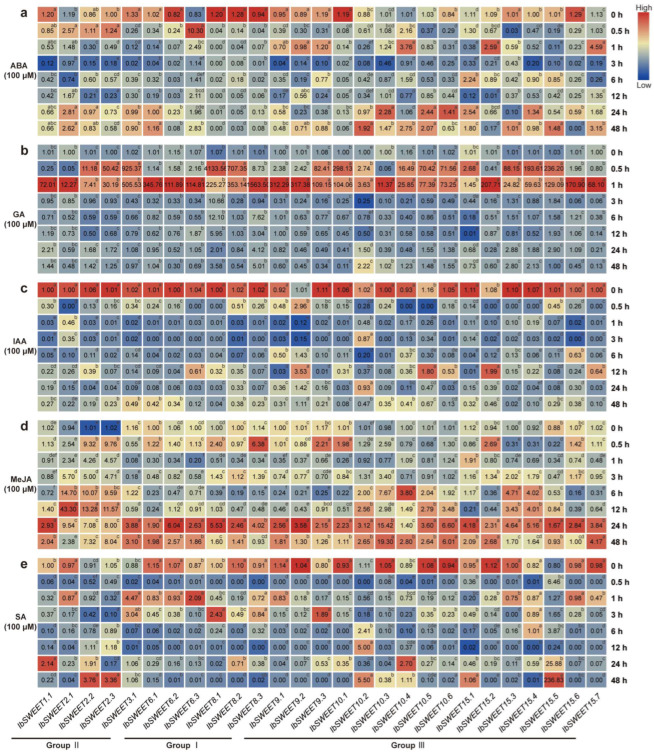
Gene expression patterns of *IbSWEETs* in response to different phytohormones ((**a**) ABA, (**b**) GA, (**c**) IAA, (**d**) MeJA, and (**e**) SA) of *I. batatas.* The values were determined by qRT-PCR from three biological replicates consisting of pools of three plants, and the results were analyzed using the comparative C_T_ method. The expression of 0 h in each treatment was considered as “1”. The fold changes are shown in the boxes. Different lowercase letters indicate a significant difference of each *IbSWEET* at *p <* 0.05 based on Student’s *t*-test.

To investigate the potential biological functions of *IbSWEETs* in the hormone signal transduction and crosstalk of plants, we investigated the expressions of SWEETs under various hormonal treatments in order to explore the relationships between SWEETs and hormones. We performed qRT-PCR to evaluate the expression levels of *IbSWEETs* in response to hormones, including ABA, GA, IAA, MeJA, and SA (Figure 10). Under ABA treatment, *IbSWEET6.3* (10.30-fold), *IbSWEET10.4* (3.76-fold), and *IbSWEET15.7* (4.59-fold) were highly induced (Figure 10a). Under GA treatment, all of the *IbSWEETs* were strongly induced at 0.5 or 1 h (Figure 10b). Under IAA treatment, most of the *IbSWEETs* were repressed, except *IbSWEET9.2,-10.5*, and *-15.2* (Figure 10c). Under MeJA, most of the *IbSWEETs* were induced after 24 h. *IbSWEET2.1,-2.2*, and *-2.3* were induced by MeJA at all of the time points (Figure 10d). Under SA treatment, most of the *IbSWEETs* were sharply repressed at 0.5 h but induced at other time points (Figure 10e). These results indicate that *IbSWEETs* are differentially expressed in response to various types of hormone induction and that they participate in the crosstalk between various hormones.

**Figure 11:**
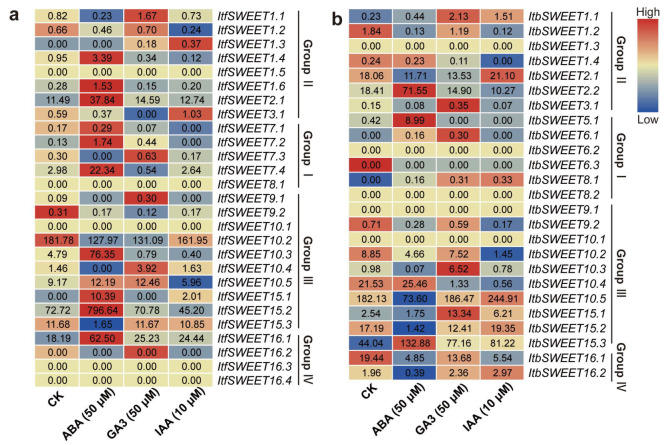
Gene expression patterns of SWEETs in response to different phytohormones (ABA, IAA, and GA) in *I. trifida* (**a**) and *I. triloba* (**b**) as determined by RNA-seq. The log_2_(FPKM+1) value is shown in the boxes.

In addition, we analyzed the expression patterns of *ItfSWEETs* and *ItbSWEETs* using the RNA-seq data of *I.trifida* and *I.triloba* under ABA, GA, and IAA treatments. In *I. trifida, ItfSWEET1.4, -1.6, -2.1, -7.1, -7.2, -7.4, -10.3, -10.5, 15.1, -15.2*, and *-16.1* were induced by ABA. *ItfSWEET1.1, -1.3, -7.2, -7.3, -9.1, -10.4, -10.5*, and *-16.1* were induced by GA3. *ItfSWEET1.3,-3.1*, and *-15.1* were induced by IAA. *ItfSWEET16.1* was induced by all the hormones, but *ItfSWEET9.2* and *ItfSWEET10.2* were repressed by all the hormones (Figure 11). In *I.triloba*, the *ItbSWEETs* showed expression patterns that differed from the homologous gene in *I. trifida. ItbSWEET2.2,-5.1,-6.1*, and *-15.3* were induced by ABA. *ItbSWEET1.1,-1.2,-3.1,-6.1,-8.1,-10.3,-15.1*, and *-15.3* were induced by GA3. *ItbSWEET1.1, -2.1,-8.1,-10.5, -15.1,* and *-15.3* were induced by IAA. *ItbSWEET15.3* was induced by all the treatments, but *ItbSWEET1.2,-9.2,-10.2*, and *-16.1* were repressed under all the hormone treatments (Figure 11). These results indicate that *SWEETs* are involved in different hormonal pathways in the sweet potato and its two diploid relatives.

#### 2.6.5. Expression Analysis under Abiotic Stresses

**Figure 12:**
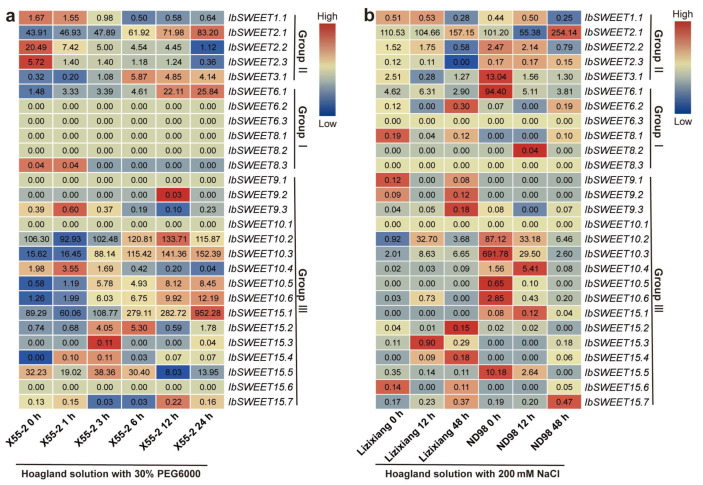
Gene expression patterns of *IbSWEETs* under drought and salt stresses as determined by RNA-seq. (**a**) Expression analysis of *IbSWEETs* under PEG treatment in a drought-tolerant variety, i.e., Xu55-2. (**b**) Expression analysis of *IbSWEETs* under NaCl treatment in a salt-sensitive variety, i.e., Lizixiang, and a salt-tolerant line, i.e., ND98. The log_2_(FPKM) value is shown in the boxes.

To explore the possible roles of *IbSWEETs* in an abiotic stress response, we analyzed the expression patterns of *IbSWEETs* using the RNA-seq data of a drought-tolerant variety (Xu55-2) under drought stress, and the RNA-seq data of a salt-sensitive variety (Lizixiang) and a salt-tolerant line (ND98) under salt stress [45,46]. *IbSWEET2.1,-10.4,-15.1*, and *-15.7* were induced by both PEG and NaCl treatments in Xu55-2 and ND98 (Figure 12).

In addition, we also analyzed the expression patterns of SWEETs using the RNA-seq data of *I. trifida* and *I. triloba* under drought and salt treatments [36]. *ItfSWEET2.1,-7.4,-10.3,-10.5,-15.1,-15.2*, and *-16.2* and *ItbSWEET2.2,-5.1,-10.2,-10.4,-15.1*, and *-15.3* were induced by both drought and salt treatments (Figure S3). Taken together, these results indicate that SWEETs are differentially expressed in response to various abiotic stresses in the sweet potato and its two diploid relatives.

## 3. Discussion

Sugar transporters are major players in the distribution of photo-assimilates to various heterotrophic sink organs. SWEETs act as key sugar transporters and play a role in crop yield and quality formation, especially in tuberous-root crops [1,2,3,4,5,6,7,8]. However, the functions and transcriptional regulatory mechanisms of SWEETs remain largely unknown in sweet potato. Tuberous roots are the main tissues harvested from sweet potato, but sweet potato’s probable progenitor diploids *I.trifida* and *I. triloba* cannot form tuberous roots. Due to the complex genetic background of cultivated sweet potato, recent studies on its gene families have mainly focused on *I.trifida* and *I. triloba* [36,47,48,49]. In this study, we systematically identified SWEETs and compared their characteristics between cultivated hexaploidy sweet potato and its two diploid relatives based on their genome sequences. A genome-wide study of SWEETs is necessary to gain a better understanding of their functions and the molecular breeding of sweet potato.

### 3.1. Evolution of the SWEET Gene Family in the Sweet Potato and Its Two Diploid Relatives

In this study, a total of 79 SWEETs were identified in sweet potato and its two diploid relatives. The number of SWEETs identified in *I. batatas* (27) was the same as that in *I. trifida* (27), but there were two fewer in *I. triloba* (25) (Figure 2; Table S1). Genomic alignment revealed the differentiation and evolution of chromosomes [50]. The chromosome localization and distribution of the SWEETs in each chromosome differed between *I. batatas, I. trifida*, and *I. triloba*; 11 chromosomes contained SWEET genes in *I.batatas* and *I. triloba*, but 10 chromosomes contained SWEET genes in *I.trifida* (Figure 2). Based on the phylogenetic relationship, the SWEETs were divided into four subgroups (Group Ⅰ to Ⅳ). There were no IbSWEETs in Group Ⅲ (Figure 3). Moreover, the number and type of SWEETs distributed in each subgroup of the sweet potato and its two diploid relatives were different from those in *Arabidopsis* and other plants (Figure 3). These results reveal that the SWEET gene family might have undergone a lineage-specific differentiation event in the terrestrial plant genome.

Five conserved motifs were identified in all 79 SWEETs, and all the SWEETs were found to contain a PQ-loop, indicating that these motifs are evolutionarily conserved among the sweet potato and its two diploid relatives. In *Arabidopsis*, four SANT-domain proteins (SANT1-4) were found to form a complex with HDA6 to regulate flowering [37]. Only ItfSWEET9.1 and ItbSWEET9.1, which were highly expressed in the flower and flower bud, were found to contain a SANT domain (Figure 4b). Introns usually act as buffer zones or mutation-resistant fragments that reduce adverse mutations and insertions. Moreover, introns also play essential roles in mRNA export, transcriptional coupling, alternative splicing, gene expression regulation, and other biological processes [50,51]. Here, the exon–intron distributions of some homologous SWEETs were different in *I. batatas* compared with those in *I. trifida* and *I. triloba* (Figure 4c). For example, in Group I, *IbSWEET8.1* contained one intron, but its homologous genes, *ItfSWEET8.1* and *ItbSWEET8.1,* contained five introns. In Group III, *IbSWEET15.1, ItfSWEET15.1*, and *ItbSWEET15.1* contained six, four, and six exons, respectively. In the sweet potato and the two diploids, these differences in the exon–intron structure may result in the different functions carried out by SWEETs in plant development [52,53,54].

### 3.2. Different Functions of SWEETs in Tuberous Root Development in Sweet Potato

In plants, SWEETs have been reported to be involved in root development and assimilate accumulation. The *atsweet11* and *atsweet12* double mutants exhibited delayed root development and severe modifications to the chemical composition of the xylem cell wall [19]. The knockout of *OsSWEET11* significantly decreased the sucrose concentration in mutant embryo sacs and led to defective grain filling [27,55]. For the sweet potato, the formation and development of tuberous roots is critical to the roots’ yield and quality. Storage-root formation has been considered to be a process of assimilate accumulation [56]. As major transporters governing long-distance transport and sugar accumulation in sink cells, SWEETs may play vital roles in tuberous root development in the sweet potato [12,57]. In this study, most *IbSWEETs* peaked during the initial development stage (20 d) and the rapid expansion stage (50 d) of the tuberous roots, respectively (Figure 8). These results indicate that *IbSWEETs* may participate in tuberous root formation by regulating assimilate accumulation in sweet potato.

The flesh color of the tuberous root is one of the most important quality characteristics of the sweet potato. Most of the *IbSWEETs* were highly expressed in the yellow-fleshed varieties, which are rich in carotenoids (Figure 9). Carotenoids are derived from two isoprene isomers, isopentenyl diphosphate (IPP) and its allylic isomer, dimethylallyl diphosphate (DMAPP). IPP and DMAPP come from the Calvin–Benson cycle by fixed carbon [58,59]. Additionally, SWEETs’ transport of sucrose is a key step for fixed-carbon transport in the phloem; thus, they may provide a sufficient precursor substance for carotenoid production in the sweet potato [11,60,61]. These data indicate that *IbSWEETs* may be involved in carotenoid accumulation in sweet potato tuberous roots by transporting photo-assimilates. However, further study is required to underlie the regulatory mechanisms of SWEETs on tuberous root development and carotenoids accumulation.

### 3.3. Different Functions of SWEETs in Hormone Crosstalk in the Sweet Potato and Its Two Diploid Relatives

*SWEETs* have been reported to participate in the regulation of multiple hormones. The interaction between SWEETs and CWINV (cell wall invertase), which encodes an enzyme that catalyzes the hydrolysis of sucrose into glucose and fructose, may lead to the loss of apical dominance and the appearance of multiple shoots under cytokinins [62]. The *atsweet13* and *atsweet14* double mutant line showed function loss in transporting exogenous GA [24,25,26]. OsSWEET13a was found to be involved in the transport of GA to young leaves during the early developmental stage [24]. The overexpression of *OsSWEET5* inhibited auxin concentration, signaling, and translocation in rice [25]. In this study, each *IbSWEET* gene could be induced by at least two hormones. *IbSWEET2.1*, which contained an ABA-responsive element (i.e., ABRE, and an SA-responsive element, or the TCA motif), was induced by ABA, GA, and MeJA but repressed by IAA and SA. However, *ItbSWEET2.1* was induced by IAA, and there was no significant change in ItfSWEET2.1 under IAA treatment. *IbSWEET8.1*, which contained a TCA motif, was induced by GA, MeJA, and SA but repressed by ABA and IAA treatments (Figure 10). However, *ItbSWEET8.1* was induced by IAA. *IbSWEET15.5*, which contained a GA-responsive element (i.e., the TATC-box, and JA-responsive elements, or a TGACG motif, an ABRE, and a TCA motif), was significantly induced by GA and SA. *IbSWEET15.3*, which contained a TGACG motif and an ABRE was repressed under ABA treatment, but *ItbSWEET15.3* was induced by ABA, GA, and IAA. *ItbSWEET16.1* was repressed under ABA treatment, but *ItfSWEET16.1* was induced by ABA (Figure 11). These results indicate that SWEETs are involved in the crosstalk of multiple hormones and that homologous SWEET genes participate in different hormone pathways in sweet potato and its two diploid relatives (Tables S2 and S3). However, the roles of SWEETs in the regulation of hormone crosstalk still need further investigation.

### 3.4. Different Functions of SWEETs in Abiotic Stress Response in the Sweet Potato and Its Two Diploid Relatives

*SWEETs* have been reported to participate in the abiotic stress response in plants. In grapes, *VvSWEET11* and *VvSWEET15* were found to be significantly induced by heat treatment [63]. In Arabidopsis, *AtSWEET15* was highly expressed under cold and salinity treatments [64]. Here, SWEETs were differentially expressed in response to various abiotic stresses in the sweet potato and its two diploid relatives. In the sweet potato, *IbSWEET2.1, -10.4, -15.1,* and *-15.7* were induced by both PEG and NaCl treatments in Xu55-2 and ND98 (Figure 12). Moreover, the diploids *I. trifida* and *I. triloba* could be used to discover functional genes, particularly genes conferring resistance or tolerance to biotic and abiotic stress, which were possibly lost in the cultivated sweet potato during its domestication [57]. In the two diploid relatives, *ItfSWEET2.1,-7.4, -10.3, -10.5, -15.1, -15.2,* and *-16.2* and *ItbSWEET2.2, -5.1, -10.2, -10.4, -15.1,* and *-15.3* were induced by both drought and salt treatments (Figure S3). These SWEETs may serve as candidate genes for use in improving abiotic stress tolerance in sweet potato.

## 4. Materials and Methods

### 4.1. Identification of SWEETs

The whole-genome sequences of *I. batatas, I. trifida,* and *I. triloba* were downloaded from the *Ipomoea* Genome Hub (https://ipomoea-genome.org/, accessed on 26 July 2022) and the Sweetpotato Genomics Resource (http://sweetpotato.plantbiology.msu.edu/, accessed on 26 July 2022). To accurately identify all the SWEET family members, three different screening methods were combined. First, the BLAST algorithm was used to identify the predicted SWEETs using all the *AtSWEETs* from the *Arabidopsis* genome database (https://www.arabidopsis.org/, accessed on 27 July 2022) as queries (BLASTP, E value ≤ 1 × 10^−5^). Next, the HMMER 3.0 software was used to identify potential SWEETs through the Hidden Markov Model profiles (hmmsearch, E value ≤ 1 × 10^−5^) of the PQ-loop domain (pfam04193), which were extracted from the Pfam databases (http://pfam.xfam.org/, accessed on 27 July 2022). Finally, all the putative SWEETs were ensured using SMART (http://smart.embl-heidelberg.de/, accessed on 27 July 2022) and CD-search (https://www.ncbi.nlm.nih.gov/Structure/cdd/wrpsb.cgi, accessed on 27 July 2022).

### 4.2. Chromosomal Distribution of SWEETs

The *IbSWEETs, ItfSWEETs*, and *ItbSWEETs* were separately mapped to the *I. batatas, I. trifida,* and *I. triloba* chromosomes, respectively, based on the chromosomal locations provided in the *Ipomoea* Genome Hub (https://ipomoea-genome.org/, accessed on 2 August 2022) and Sweetpotato Genomics Resource (http://sweetpotato.plantbiology.msu.edu/, accessed on 2 August 2022). The visualization was generated using the TBtools software (v.1.098696) (South China Agricultural University, Guangzhou, China) [65].

### 4.3. Protein Properties Prediction of SWEETs

The MW, theoretical pI, unstable index, and hydrophilic of the SWEETs were calculated using ExPASy (https://www.expasy.org/, accessed on 4 August 2022). The phosphorylation sites of the SWEETs were predicted using GPS 5.0 [66]. The subcellular localization of the SWEETs was predicted using Plant-mPLoc (http://www.csbio.sjtu.edu.cn/bioinf/plant-multi/, accessed on 4 August 2022). The TMHs of the SWEETs were predicted using TMHMM-2.0 (https://services.healthtech.dtu.dk/service.php?TMHMM-2.0, accessed on 4 August 2022). The 3D structural model of the SWEETs was built using SWISS-MODEL (https://swissmodel.expasy.org/, accessed on 4 August 2022) [67]

### 4.4. Phylogenetic Analysis of SWEETs

Multiple sequence alignment of the deduced amino acid sequences of the SWEETs from *I. batatas, I. trifida, I. triloba, Arabidopsis, Zea mays*, and *Oryza sativa* were aligned with Clustal X, and the alignment was imported into MEGA11 to create a phylogenetic tree using the neighbor-joining method with 1000 bootstrap replicates (www.megasoftware.net, accessed on 3 December 2022) [68]. Then, the phylogenetic tree was constructed using iTOL (http://itol.embl.de/, accessed on 3 December 2022).

### 4.5. Domain Identification and Conserved Motif Analysis of SWEETs

The conserved motifs of the SWEETs were analyzed using MEME software (https://meme-suite.org/meme/, accessed on 5 August 2022). The MEME parameters were set to search for a maximum of 15 motifs with a motif width comprised between 5 and 50 residues [69].

### 4.6. Exon–Intron Structures and Promoter Analysis of SWEETs

The exon–intron structures of the SWEETs were obtained from GSDS 2.0 (http://gsds.gao-lab.org/, accessed on 6 August 2022) and were visualized using the TBtools software. The *cis*-elements in the approximately 1500 bp promoter region of the SWEETs were predicted using PlantCARE (http://bioinformatics.psb.ugent.be/webtools/plantcare/html/, accessed on 6 August 2022) [70].

### 4.7. Protein Interaction Network of SWEETs

The protein interaction networks of the SWEETs were predicted using STRING (https://cn.string-db.org/, accessed on 7 August 2022) based on *Arabidopsis* homologous proteins. The network map was built using Cytoscape software [71].

### 4.8. qRT-PCR Analysis of SWEETs

The salt-tolerant sweet potato (*I. batatas*) line ND98 was used for qRT-PCR analysis in this study [45]. In vitro grown ND98 plants were cultured on Murashige and Skoog (MS) medium at 27 ± 1 °C under a photoperiod consisting of 13 h of cool-white fluorescent light at 54 μmol m^−2^ s^−1^ and 11 h of darkness. The sweet potato plants were cultivated in a field in the campus of China Agricultural University, Beijing, China.

For expression analysis in various tissues, the total RNA was extracted from the buds, leaves, petioles, stems, pencil roots, and tuberous root tissues of 3-month-old field-grown ND98 plants; the different development stage of the tuberous root tissues of Y25 (3 d, 10 d, 20 d, 30 d, 40 d, 50 d, 60 d, 70 d, 80 d, and 90 d) and the tuberous root tissues of different field-grown plants at 90 d (Jiyuan3, Shangshu19, Longshu9, Yanshu32, Luozi5, and Qin12-20-11) were analyzed using the TRIzol method (Invitrogen). For the expression analysis of the hormone treatment, the leaves were sampled at 0, 0.5, 1, 3, 6, 12, 24, and 48 h after being treated with 100 μM ABA, 100 μM GA, 100 μM IAA, 100 μM MeJA, and 100 μM SA, respectively. Three independent biological replicates were taken, each with three plants. qRT-PCR was conducted using the SYBR detection protocol (TaKaRa, Kyoto, Japan) on a 7500 Real-Time PCR system (Applied Biosystems, Foster City, CA, USA). The reaction mixture was composed of first-strand cDNA, a primer mix, and an SYBR Green M Mix (TaKaRa; code RR420A) with a final volume of 20 μL. A sweet potato actin gene (GenBank AY905538) was used as an internal control. The relative gene expression levels were quantified using the comparative C_T_ method [72]. The specific primers used for the qRT-PCR analysis are listed in Table S4. The heat maps of the gene expression profiles were constructed using the TBtools software (v.1.098696) [65].

### 4.9. Transcriptome Analysis

The RNA-seq data of *ItfSWEETs* and *ItbSWEETs* in *I. trifida* and *I. triloba* were downloaded from the Sweetpotato Genomics Resource (http://sweetpotato.plantbiology.msu.edu/, accessed on 10 August 2022). The RNA-seq data of *IbSWEETs* in *I. batatas* were obtained from the NCBI SRA repository under the accession number SRP092215 [45,46]. The expression levels of the SWEETs were calculated as fragments per kilobase of exon per million fragments mapped (FPKM). The heat maps were constructed using the Tbtools software (v.1.098696) [65].

## 5. Conclusions

In this study, we identified and characterized 27, 27, and 25 SWEETs in cultivated hexaploidy sweet potato (*I. batatas*, 2n = 6x = 90) and its two diploid relatives, *I. trifida* (2n = 2x = 30) and *I. triloba* (2n = 2x = 30), respectively, based on genome and transcriptome data. The protein physicochemical properties, chromosome localization, phylogenetic relationships, gene structures, promoter *cis*-elements, and protein interaction networks of these 79 SWEETs were systematically investigated. Moreover, the tissue specificity and expression patterns of the SWEETs in tuberous root development, hormone responses, and abiotic stress responses were analyzed using qRT-PCR and RNA-seq. The results indicated that there was a differentiation in the functions of homologous SWEETs in the sweet potato and its two diploid relatives, and each SWEET gene played different vital roles in the plants’ growth and development, carotenoid accumulation, hormone crosstalk, and abiotic stress response. This study provides valuable insights into the structure and function of SWEET genes in the sweet potato and its two diploid relatives.

## Figures and Tables

**Table 1:** Characterization of *IbSWEETs* in sweet potato.

Gene ID	Gene Name	PI	MW/kDa	Genomic Length/bp	CDS Length/bp	Phosphorylation Site			Protein Size/aa	Aliphatic Index	GRAVY	TMHs	Subcellular Locations	*Arabidopsis* Homologous
						Ser	Thr	Tyr						
g42355	*IbSWEET1.1*	9.55	27.63	1949	1158	17	12	6	254	120.47	0.819	7	Cell membrane Golgi apparatus	*SWEET1*
g45970	*IbSWEET2.1*	9.18	30.50	2865	1303	30	11	12	273	114.58	0.788	7	Cell membrane	*SWEET2*
g37512	*IbSWEET2.2*	8.97	26.17	2620	1086	23	12	14	235	125.19	1.003	7	Cell membrane	*SWEET2*
g37574	*IbSWEET2.3*	9.44	19.99	4204	1557	17	11	9	179	125.70	1.070	5	Cell membrane	*SWEET2*
g20639	*IbSWEET3.1*	8.83	24.44	1825	829	20	12	10	217	98.25	0.281	5	Cell membrane	*SWEET3*
g39263	*IbSWEET6.1*	8.46	30.93	2934	1046	19	17	12	278	126.19	0.871	7	Cell membrane	*SWEET6*
g39260	*IbSWEET6.2*	9.15	25.53	2101	868	19	16	11	233	105.41	0.481	5	Cell membrane	*SWEET6*
g39262	*IbSWEET6.3*	9.30	25.79	2900	983	22	15	11	237	112.32	0.523	6	Cell membrane	*SWEET6*
g5800	*IbSWEET8.1*	9.83	22.47	1052	966	15	10	7	206	117.86	0.639	6	Cell membrane	*SWEET8*
g346	*IbSWEET8.2*	9.47	25.72	1977	1065	17	11	10	235	120.68	0.681	7	Cell membrane	*SWEET8*
g51687	*IbSWEET8.3*	9.98	26.48	2536	1055	16	14	7	239	108.20	0.592	6	Cell membrane	*SWEET8*
g41769	*IbSWEET9.1*	9.16	27.26	1912	823	12	7	14	241	119.71	0.747	7	Cell membrane	*SWEET9*
g49942	*IbSWEET9.2*	9.48	30.39	5035	1049	15	14	17	267	114.68	0.696	6	Cell membrane	*SWEET9*
g33162	*IbSWEET9.3*	8.72	30.49	2028	1395	16	22	13	275	122.15	0.691	6	Cell membrane	*SWEET9*
g6315	*IbSWEET10.1*	8.83	31.13	2310	1122	16	16	14	278	117.73	0.700	7	Cell membrane	*SWEET10*
g33248	*IbSWEET10.2*	9.34	34.07	3208	1235	17	18	11	305	114.72	0.549	7	Cell membrane	*SWEET10*
g55355	*IbSWEET10.3*	9.20	34.65	1851	1231	18	11	13	314	122.26	0.689	7	Cell membrane	*SWEET10*
g38390	*IbSWEET10.4*	9.19	34.25	2664	1264	21	17	11	304	117.57	0.607	7	Cell membrane	*SWEET10*
g14486	*IbSWEET10.5*	9.48	30.78	3130	1123	18	15	9	272	106.76	0.521	5	Cell membrane	*SWEET10*
g14649	*IbSWEET10.6*	9.39	32.65	3831	1188	17	17	11	288	116.39	0.678	7	Cell membrane	*SWEET10*
g4174	*IbSWEET15.1*	7.61	35.41	2008	1238	19	19	11	321	114.70	0.568	7	Cell membrane	*SWEET15*
g39828	*IbSWEET15.2*	8.19	33.64	2933	1057	19	16	12	302	115.79	0.541	6	Cell membrane	*SWEET15*
g13599	*IbSWEET15.3*	9.46	24.64	1780	896	16	10	8	221	127.87	0.802	6	Cell membrane	*SWEET15*
g13600	*IbSWEET15.4*	9.30	24.86	1917	920	19	12	8	222	124.19	0.821	6	Cell membrane	*SWEET15*
g13601	*IbSWEET15.5*	7.74	32.80	1767	1103	24	9	11	292	120.17	0.664	7	Cell membrane	*SWEET15*
g61464	*IbSWEET15.6*	5.81	31.87	1594	1026	29	13	9	278	119.10	0.729	4	Cell membrane Chloroplast	*SWEET15*
g61461	*IbSWEET15.7*	9.47	17.64	5747	988	14	9	7	153	127.97	0.907	6	Cell membrane Chloroplast	*SWEET15*

CDS, coding sequence; MW, molecular weight; pI, isoelectric point; Ser, serine; Thr, threonine; Tyr, tyrosine; TMHs, transmembra-ne helices.

## Data Availability

The data presented in this study are available on request from the corresponding author.

## Supplementary Materials

The supporting information can be downloaded at: https://www.mdpi.com/article/10.3390/ijms232415848/s1.
