# Supplementary material for: Genome-Wide Identification and Expression Analysis of SWEET Family Genes in Sweet Potato and Its Two Diploid Relatives
Source: Int J Mol Sci. 2022 Dec 13;23(24):15848. doi: 10.3390/ijms232415848 (PMC9785306; doi:10.3390/ijms232415848)
Supplement: Supplementary file 1 [file ijms-23-15848-s001.zip › ijms-1929186-supplementary.pdf]

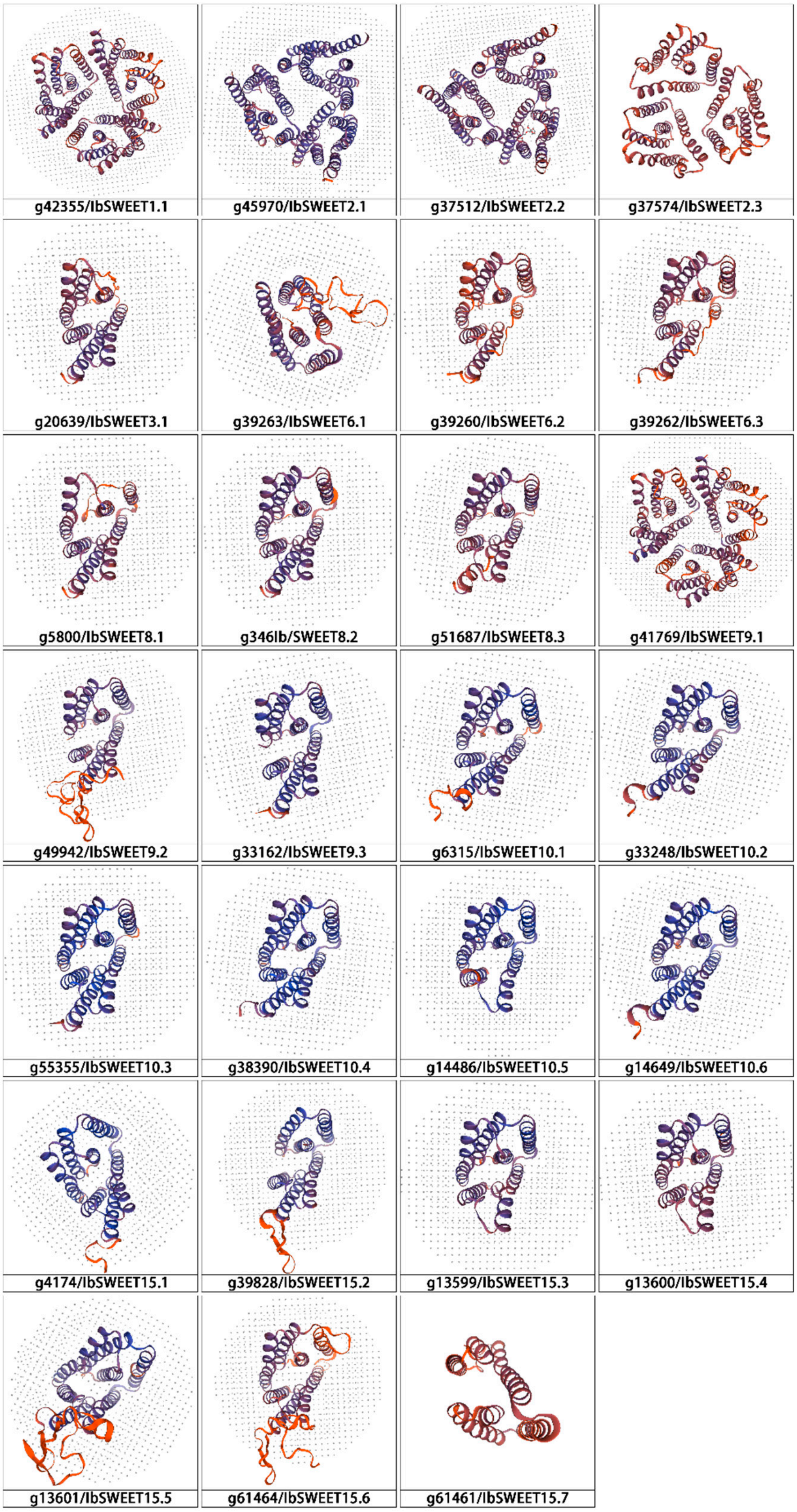

Figure S1. The three-dimensional structural models of IbSWEETs in *I. batatas*

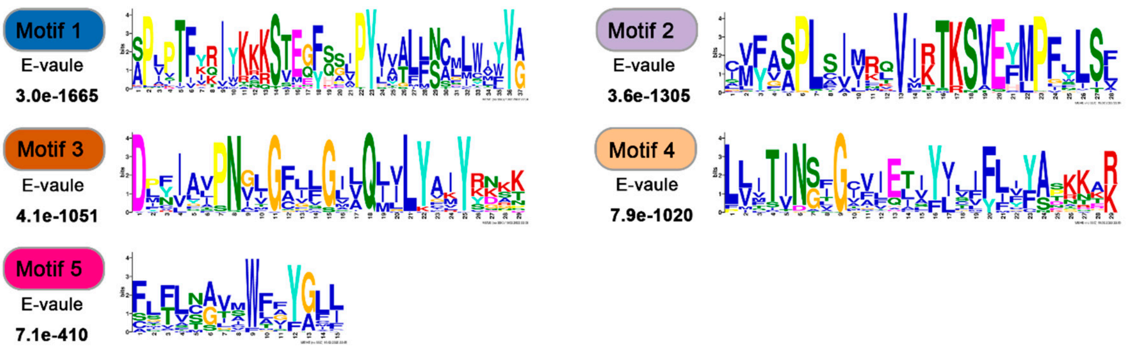

Figure S2. Conserved motifs analysis of IbSWEETs in *I. batatas*.

The Gene expression patterns of ItfSWEETs in response to different phytohormones;

The Gene expression patterns of ItbSWEETs in response to different phytohormones

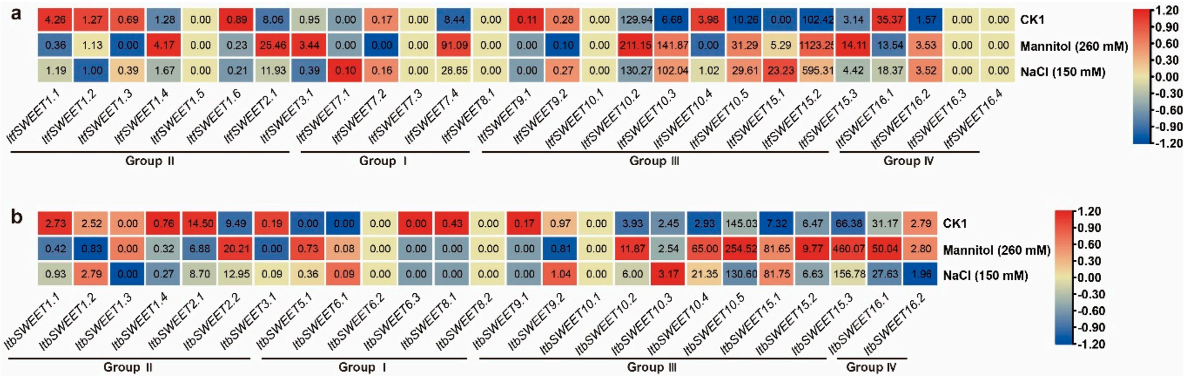

Figure S3. Gene expression patterns of SWEETs in response to different abiotic stress (Mannitol and NaCl) in *I. trifida* and *I. triloba* as determined by RNA-seq. Log2 (FPKM+1) was shown in the boxes.

Tabel S1. Identification of *SWEET* family genes in *I. batatas*, *I. trifida*, and *I. triloba*.

Arabidopsis	**Homologous gene in *I.batatas*/*I. trifida*/ *I. triloba***	**Gene ID**	**Gene name**	**Chromosome localization**
Group Ⅰ： *AT3G28007.1/AtSWEET4* *AT5G62850.1/AtSWEET5* *AT1G66770.1/AtSWEET6* *AT4G10850.1/AtSWEET7* *AT5G40260.1/AtSWEET8*	*I. batatas*	*g39263*	*IbSWEET6.1*	*LG10:7838441-7841375*
		g39260	*IbSWEET6.2*	*LG10:7816324-7818425*
		g39262	*IbSWEET6.3*	*LG10:7830865-7833765*
		g5800	*IbSWEET8.1*	*LG2:10784966-10786018*
		g346	*IbSWEET8.2*	*LG1:1808926-1810903*
		g51687	*IbSWEET8.3*	*LG13:3782061-3784597*
	I. trifida	*itf12g17160.t1*	*ItfSWEET7.1*	*Chr12:16815989-16818214*
		itf08g07870.t1	*ItfSWEET7.2*	*Chr08:5841213-5846012*
		itf08g07860.t1	*ItfSWEET7.3*	*Chr08:5831106-5834414*
		itf08g07850.t2	*ItfSWEET7.4*	*Chr08:5814193-5817068*
		itf05g24560.t1	*ItfSWEET8.1*	*Chr05:24311908-24313582*
	I. triloba	*itb02g13210.t1*	*ItbSWEET5.1*	*Chr02:9244237-9246292*
		itb12g17890.t1	*ItbSWEET6.1*	*Chr12:20145343-20147563*
		itb08g08270.t1	*ItbSWEET6.2*	*Chr08:7215664-7220968*
		itb08g08260.t1	*ItbSWEET6.3*	*Chr08:7204389-7207714*
		itb02g23980.t1	*ItbSWEET8.1*	*Chr02:24313432-24315665*
		itb05g25360.t1	*ItbSWEET8.2*	*Chr05:29797696-29799315*
Group Ⅱ： *AT1G21460.1/AtSWEET1* *AT3G14770.1/AtSWEET2* *AT5G53190.1/AtSWEET3*	*I.batatas*	*g42355*	*IbSWEET1.1*	*LG11:6571044-6572993*
		g45970	*IbSWEET2.1*	*LG11:34025564-34028429*
		g37512	*IbSWEET2.2*	*LG9:26404546-26407166*
		g37574	*IbSWEET2.3*	*LG9:26774560-26778764*
		g20639	*IbSWEET3.1*	*LG5:29266168-29267993*
	I. trifida	*itf01g30090.t1*	*ItfSWEET1.1*	*Chr01:28489958-28492016*
		itf06g14370.t1	*ItfSWEET1.2*	*Chr06:17600146-17603483*
		itf05g11210.t1	*ItfSWEET1.3*	*Chr05:13456959-13464258*
		itf05g11180.t1	*ItfSWEET1.4*	*Chr05:13426791-13433075*
		itf05g10340.t1	*ItfSWEET1.5*	*Chr05:12171409-12175054*
		itf05g11190.t1	*ItfSWEET1.6*	*Chr05:13438573-13447544*
		itf01g06490.t1	*ItfSWEET2.1*	*Chr01:4604929-4607829*
		itf12g25940.t1	*ItfSWEET3.1*	*Chr12:23179604-23181318*
	I. triloba	*itb01g29890.t1*	*ItbSWEET1.1*	*Chr01:33995339-33997526*
		itb06g12580.t1	*ItbSWEET1.2*	*Chr06:17231093-17234294*
		itb05g11560.t1	*ItbSWEET1.3*	*Chr05:17666984-17675087*
		itb05g11570.t1	*ItbSWEET1.4*	*Chr05:17686534-17694593*
		itb10g03630.t1	*ItbSWEET2.1*	*Chr10:3402029-3404651*
		itb01g07080.t1	*ItbSWEET2.2*	*Chr01:5276517-5279474*
		itb12g26330.t1	*ItbSWEET3.1*	*Chr12:27293008-27295110*
	I.batatas	*g41769*	*IbSWEET9.1*	*LG11:2589559-2591471*
		g49942	*IbSWEET9.2*	*LG12:22929904-22934939*

Group Ⅲ:<br>*AT2G39060.1/AtSWEET9*<br>*AT5G50790.1/AtSWEET10*<br>*AT3G48740.1/AtSWEET11*<br>*AT5G23660.1/AtSWEET12*<br>*AT5G50800.1/AtSWEET13*<br>*AT4G25010.1/AtSWEET14*<br>*AT5G13170.1/AtSWEET15*		*g33162*	*IbSWEET9.3*	*LG8:20422984-20425012*
		g6315	*IbSWEET10.1*	*LG2:14785339-14787649*
		g33248	*IbSWEET10.2*	*LG8:21098280-21101488*
		g55355	*IbSWEET10.3*	*LG13:29379559-29381410*
		g38390	*IbSWEET10.4*	*LG10:1596906-1599570*
		g14486	*IbSWEET10.5*	*LG4:12551915-12555045*
		g14649	*IbSWEET10.6*	*LG4:13860424-13864255*
		g4174	*IbSWEET15.1*	*LG1:30131225-30133233*
		g39828	*IbSWEET15.2*	*LG10:12111176-12114109*
		g13599	*IbSWEET15.3*	*LG4:5491975-5493755*
		g13600	*IbSWEET15.4*	*LG4:5496388-5498305*
		g13601	*IbSWEET15.5*	*LG4:5501178-5502945*
		g61464	*IbSWEET15.6*	*LG15:10703749-10705343*
		g61461	*IbSWEET15.7*	*LG15:10669805-10675552*
	I. trifida	*itf01g34820.t1*	*ItfSWEET9.1*	*Chr01:31439366-31444973*
		itf11g15210.t1	*ItfSWEET9.2*	*Chr11:11572968-11575147*
		itf04g17080.t1	*ItfSWEET10.1*	*Chr04:16136022-16138460*
		itf11g18410.t1	*ItfSWEET10.2*	*Chr11:15683610-15687029*
		itf02g04770.t1	*ItfSWEET10.3*	*Chr02:5937857-5939964*
		itf08g02230.t1	*ItfSWEET10.4*	*Chr08:1554537-1557236*
		itf13g10090.t1	*ItfSWEET10.5*	*Chr13:12482270-12485935*
		itf13g17000.t2	*ItfSWEET15.1*	*Chr13:19113650-19115066*
		itf05g00920.t1	*ItfSWEET15.2*	*Chr05:737228-739500*
		itf08g10570.t1	*ItfSWEET15.3*	*Chr08:8975012-8978094*
	I. triloba	*itb01g34620.t1*	*ItbSWEET9.1*	*Chr01:37215717-37221099*
		itb11g16570.t1	*ItbSWEET9.2*	*Chr11:14815811-14817710*
		itb04g18880.t1	*ItbSWEET10.1*	*Chr04:22666706-22668974*
		itb13g13530.t1	*ItbSWEET10.2*	*Chr13:20041045-20044131*
		itb08g02250.t1	*ItbSWEET10.3*	*Chr08:1811788-1814606*
		itb02g00080.t1	*ItbSWEET10.4*	*Chr02:66527-68913*
		itb11g20660.t1	*ItbSWEET10.5*	*Chr11:21859398-21862619*
		itb13g20410.t1	*ItbSWEET15.1*	*Chr15:27222781-27224625*
		itb08g11840.t1	*ItbSWEET15.2*	*Chr08:11826103-11828754*
		itb05g00340.t1	*ItbSWEET15.3*	*Chr05:266867-269193*
Group Ⅳ:<br>*AT3G16690.1/AtSWEET16*<br>*AT4G15920.1/AtSWEET17*	*I.batatas*			
	I. trifida	*itf04g18250.t1*	*ItfSWEET16.1*	*Chr04:18378337-18384219*
		itf15g13030.t1	*ItfSWEET16.2*	*Chr15:9811883-9815347*
		itf15g13020.t1	*ItfSWEET16.3*	*Chr15:9799924-9802933*
		itf15g13040.t1	*ItfSWEET16.4*	*Chr15:9819185-9827065*
	I. triloba	*itb04g18030.t1*	*ItbSWEET16.1*	*Chr04:21289222-21296312*
		itb15g13360.t1	*ItbSWEET16.2*	*Chr15:11360246-11362622*

Table S2 The Responses of *IbSWEETs* to hormones.

ID		ABA	GA	IAA	MeJA	SA
IbSWEET1.1	Group Ⅱ	repressed	induced	repressed	induced	induced
IbSWEET2.1		induced	induced	repressed	induced	repressed
IbSWEET2.2		repressed	induced	repressed	induced	induced
IbSWEET2.3		induced	induced	repressed	induced	induced
IbSWEET3.1		repressed	induced	repressed	induced	induced
IbSWEET6.1	Group Ⅰ	repressed	induced	repressed	induced	repressed
IbSWEET6.2		repressed	induced	repressed	induced	repressed
IbSWEET6.3		induced	induced	repressed	induced	induced
IbSWEET8.1		repressed	induced	repressed	induced	induced
IbSWEET8.2		repressed	induced	repressed	induced	repressed
IbSWEET8.3		repressed	induced	repressed	induced	repressed
IbSWEET9.1	Group Ⅲ	repressed	induced	repressed	induced	repressed
IbSWEET9.2		repressed	induced	induced	induced	repressed
IbSWEET9.3		repressed	induced	repressed	induced	induced
IbSWEET10.1		repressed	induced	repressed	induced	repressed
IbSWEET10.2		induced	induced	repressed	induced	induced
IbSWEET10.3		induced	induced	repressed	induced	repressed
IbSWEET10.4		induced	induced	repressed	induced	induced
IbSWEET10.5		induced	induced	induced	induced	repressed
IbSWEET10.6		induced	induced	repressed	induced	repressed
IbSWEET15.1		induced	induced	repressed	induced	repressed
IbSWEET15.2		induced	induced	induced	induced	repressed
IbSWEET15.3		repressed	induced	repressed	induced	repressed
IbSWEET15.4		induced	induced	repressed	induced	repressed
IbSWEET15.5		induced	induced	repressed	induced	induced
IbSWEET15.6		repressed	induced	repressed	induced	repressed
IbSWEET15.7		induced	induced	repressed	induced	repressed

Table S3 The Responses of *ItfSWEETs* and *ItbSWEETs* to hormones.

ID	Groups	ABA	GA3	IAA
ItfSWEET1.1	Group Ⅱ	repressed	induced	repressed
ItfSWEET1.2		repressed	induced	repressed
ItfSWEET1.3		-	induced	induced
ItfSWEET1.4		induced	repressed	repressed
ItfSWEET1.5		-	-	-
ItfSWEET1.6		induced	repressed	repressed
ItfSWEET2.1		induced	induced	-
ItfSWEET3.1		-	repressed	induced
ItfSWEET7.1	Group Ⅰ	induced	repressed	-
ItfSWEET7.2		induced	induced	-
ItfSWEET7.3		repressed	induced	repressed
ItfSWEET7.4		induced	repressed	repressed
ItfSWEET8.1		-	-	-
ItfSWEET9.1	Group Ⅲ	-	induced	repressed
ItfSWEET9.2		-	repressed	repressed
ItfSWEET10.1		-	-	-
ItfSWEET10.2		repressed	repressed	repressed
ItfSWEET10.3		induced	repressed	repressed
ItfSWEET10.4		repressed	induced	repressed
ItfSWEET10.5		induced	induced	repressed
ItfSWEET15.1		induced	-	induced
ItfSWEET15.2		induced	repressed	repressed
ItfSWEET15.3		repressed	-	-
ItfSWEET16.1	Group Ⅳ	induced	induced	induced
ItfSWEET16.2		-	-	-
ItfSWEET16.3		-	-	-
ItfSWEET16.4		-	-	-
ItbSWEET1.1	Group Ⅱ	induced	induced	induced
ItbSWEET1.2		repressed	repressed	repressed
ItbSWEET1.3		-	-	repressed
ItbSWEET1.4		-	repressed	repressed
ItbSWEET2.1		repressed	repressed	induced
ItbSWEET2.2		induced	repressed	repressed
ItbSWEET3.1		repressed	induced	repressed
ItbSWEET5.1	Group Ⅰ	induced	-	repressed
ItbSWEET6.1		induced	induced	-
ItbSWEET6.2		-	-	-
ItbSWEET6.3		-	-	-
ItbSWEET8.1		-	induced	induced
ItbSWEET8.2		-	-	-

ItbSWEET9.1	Group Ⅲ	-	-	-
ItbSWEET9.2		repressed	repressed	repressed
ItbSWEET10.1		-	-	-
ItbSWEET10.2		repressed	repressed	repressed
ItbSWEET10.3		repressed	induced	repressed
ItbSWEET10.4		induced	repressed	repressed
ItbSWEET10.5		repressed	-	induced
ItbSWEET15.1		repressed	induced	induced
ItbSWEET15.2		repressed	repressed	induced
ItbSWEET15.3		induced	induced	induced
ItbSWEET16.1	Group Ⅳ	repressed	repressed	repressed
ItbSWEET16.2		repressed	induced	induced

- indicates not found.

Table S4. Primers used in this study.

Gene	Forward Primer	Reverse Primer
IbSWEET1.1	TTTGGGTGTGGCTTAGGAGC	GCCATCAACTCCGACCATCT
IbSWEET2.1	GCCATTTTACCTCTCGCTTGC	CCGTTTGGGACGGAGATGAA
IbSWEET2.2	TTCCAGTTGGCATACCTCACC	CAAACACAGCGAAAACGCCT
IbSWEET2.3	CCAGTTGGCATACCTCACCA	CGAAAACGCCTAGCAACCAC
IbSWEET3.1	GTCGCCTCCGTGTCAATGTA	TCAGCAGCCCATAAGCCATC
IbSWEET6.1	TTGTACGCAATCCCCAGTGT	GGACGATGAGAGCAACCCTA
IbSWEET6.2	GAGGTTGTTTTCATGGCGGC	AGTGCTACCAACAATGGCAGA
IbSWEET6.3	TGCTCGTTTGTGACTGGTGT	TTGTGCCAATCCGGCTAGAG
IbSWEET8.1	GTTCGCTGTCGGTTGTGTTT	CCGCGCTCTTGCTCTTAATC
IbSWEET8.2	ATCCACACAGCGTTCTCGTC	GAGCAAACCCGCCATTTTCC
IbSWEET8.3	GACCATCAACACTCTCGGCA	TTCCCCCGAGTTTTGGCATT
IbSWEET9.1	GTTCATGCCGCTCACTCTCT	AACGATTTGGGCGATTCCGA
IbSWEET9.2	TGCAACCAAACAGGCCAAGA	TAACTCGCCTCTCACCCCTC
IbSWEET9.3	GTCGTCTGGCTTCTCTACGG	CTGCCGTTTTCCGGTCTCTA
IbSWEET10.1	CCTCAAGCCGAGCCCATATT	TGCCTCACAATGCACAATGG
IbSWEET10.2	TTCTGGGCTTCATCTTCGGG	TGCTCCTTCACTGCGTCTTT
IbSWEET10.3	AGTCCAACGCCACACTTCTC	CGCCGAAACCAACAATCACC
IbSWEET10.4	GGCTGGTTTCTCTGGTCACT	TGACTGGAACCCTTCTGTCG
IbSWEET10.5	TGGCTGCTTCATCGAAACCT	ATACCGACGCCCCAAGTAAC
IbSWEET10.6	CCCAGTTTCTGTTCAAAGGCG	TGGAGCCACAAATACGCACA
IbSWEET15.1	CGCTCCTCTTCTCATCACCA	AGAGTAGAGCCCTCCGACAA
IbSWEET15.2	GCAACCCCCGAATAACCTCA	CTCTTCGCGCACTAAAACCG
IbSWEET15.3	GCGAACAAAGCGAGGAATCA	AGGGAGAACGCCACACAAAT
IbSWEET15.4	ACGCATCAAAGAAAGCGAGG	GCGAATGCCACACAAATCCA
IbSWEET15.5	GCATCAAGTAAAGCAAGGCGT	TGCTCCCGAGAAAGGAAACC
IbSWEET15.6	TTTTCGCGGCTCATGGTTTC	AGCACTATGCCAATCGGTGA
IbSWEET15.7	TAGGAGTGTGGAGTTCTTGTCA	TCGAACCACTCCCATCATCC
Ibactin	AGCAGCATGAAGATTAAGGTTGTAGCAC	TGGAAAATTAGAAGCACTTCCTGTGAAC
